# Distinct trajectories of disease severity progression in juvenile systemic sclerosis

**DOI:** 10.1093/rheumatology/keag584

**Published:** 2026-10-20

**Authors:** Francesco Zulian, Francesca Tirelli, Biagio Castaldi, Elisabetta Zanatta, Fabio Vittadello, Greta Mastrangelo, Alessandra Meneghel

**Affiliations:** Division of Pediatric Rheumatology, Department of Women and Children Health, University of Padova, Padua, Italy; Division of Pediatric Rheumatology, Department of Women and Children Health, University of Padova, Padua, Italy; Division of Pediatric Cardiology, Department of Women and Children Health, University of Padova, Padua, Italy; Division of Rheumatology, Department of Medicine-DIMED, University of Padova, Padua, Italy; Research and Statistical Analysis, Explora, Vigodarzere, Padua, Italy; Division of Pediatric Rheumatology, Department of Women and Children Health, University of Padova, Padua, Italy; Division of Pediatric Rheumatology, Department of Women and Children Health, University of Padova, Padua, Italy

**Keywords:** disease trajectory, juvenile systemic sclerosis, latent class analysis, machine learning, outcome predictors

## Abstract

**Objective:**

Juvenile systemic sclerosis (JSSc) often presents with overlapping features, making phenotypic classification and disease progression difficult to define. This study investigated whether JSSc comprises latent disease severity subgroups with distinct longitudinal trajectories and examined baseline predictors of class membership.

**Methods:**

Retrospective cohort study using prospectively collected data from 45 patients with JSSc between January 2005 and December 2024. Demographic, clinical, laboratory and disease severity data were collected. The primary outcome was progression-free survival (PFS), defined as percentage change in the Juvenile Systemic Sclerosis Severity Score (J4S) over time. A Growth Mixture Model identified latent disease severity classes with distinct longitudinal trajectories and their baseline predictors.

**Results:**

Thirty-eight JSSc patients (66% females), followed for ≥48 months, entered the study. PFS remained high in the fibrotic and limited cutaneous JSSc subtypes (80–100%) over the observation period, whereas the diffuse and sine scleroderma subtypes showed marked deterioration, with PFS decreasing to 54% and 25%, respectively. We identified two latent classes of disease severity: Class 1 showed low severity and good treatment response, Class 2 showed an active, progressive course with poor long-term outcomes. Baseline predictors of poor outcome were J4S >7.5, treatment initiation >9 months from disease onset, and >1.5 organs involved. Latent trajectory classes showed good concordance with JSSc clinical subtypes (κ = 0.660).

**Conclusion:**

We identified two latent classes of disease severity in JSSc with clear prognostic significance. High baseline J4S, delayed treatment and greater organ involvement predicted a more severe disease course, paving the way for precision health strategies and optimized patient management.

*Rheumatology* key messages

- Juvenile systemic sclerosis (JSSc) often presents with overlapping features, making phenotypic classification and disease progression difficult to define.
- Unsupervised machine learning delineates two latent disease-severity trajectories: mild treatment-responsive and progressive high-risk disease.
- High baseline J4S, delayed treatment and a greater number of internal organs involved predict severe disease course.

## Introduction

Juvenile-onset systemic sclerosis (JSSc) is a rare, progressive, heterogeneous multisystem disease, which is characterized by autoimmune activation as well as pathognomonic tissue and vascular fibrosis. It affects ∼3 in one million children and causes life-threatening manifestations including interstitial lung disease and cardiac dysfunction [1].

Despite its rarity, JSSc carries a disproportionate burden of morbidity and, in a subset of patients, mortality, underscoring the need for reliable tools to identify, early in the disease course, those patients at greatest risk of a severe outcome.

Although it shares many core features with the adult form of systemic sclerosis (SSc), several key differences distinguish it: diffuse forms are more common than limited forms [1–4]; SSc-specific autoantibodies do not correlate with skin subtypes [5, 6]; and scleroderma sine scleroderma (ssJSSc) tends to be more aggressive than in adults [7]. Furthermore, the overall prognosis in JSSc, particularly mortality, is generally better in children [8–10].

Nowadays, predicting clinical phenotype and disease course—including which organs will be affected over time and how these influence disease progression—remains challenging [11]. Adult studies have shown associations between extension of skin involvement or specific autoantibodies and clinical phenotypes [12–16]. Anti-centromere antibodies (ACA) are linked to limited cutaneous SSc (lcSSc) and an increased risk of pulmonary arterial hypertension (PAH); anti-topoisomerase I antibodies (ATA) are associated with diffuse cutaneous SSc (dcSSc) and a higher risk of interstitial lung disease (ILD) and anti-RNA polymerase III antibodies (ARA) with rapid skin thickening and risk of renal crisis [5, 14–16].

However, even in adults, SSc often presents with overlapping, multi-organ clinical features, making subtyping difficult and not fully captured by skin or autoantibody-based classifications alone [17]. In JSSc, the clinical picture is even more complicated as the degree of skin involvement and autoantibody profile are of less help in defining disease subtypes and clinical course; therefore, early detection of at-risk individuals is difficult.

Unsupervised machine learning approaches such as Growth Mixture Modelling (GMM) offer specific advantages over conventional, clinician-defined classification in this setting: they model each patient’s full longitudinal trajectory rather than relying on cross-sectional snapshots, they accommodate within-subject correlation and missing data without requiring imputation, and they identify data-driven subgroups without presupposing group membership, potentially capturing clinically meaningful heterogeneity that skin- or autoantibody-based subtyping alone does not fully resolve.

The present study aimed to: (i) describe disease progression over time in a cohort of children with JSSc; (ii) investigate whether JSSc comprises latent subgroups of disease severity that follow distinct longitudinal trajectories; and (iii) examine baseline clinical and demographic factors that predict latent class membership.

## Methods

### Study design, patient population and data collection

We conducted a single-centre observational cohort study of consecutive children with JSSc diagnosed between January 2005 and December 2024. Children were included if fulfilled both paediatric and/or adults’ classification criteria for JSSc/SSc [18, 19] and had a minimum follow-up of four years. These correspond to the 2007 PRES/ACR/EULAR provisional classification criteria for JSSc (major criterion: proximal skin sclerosis/induration; plus ≥2 of nine categories of minor criteria) [18] and/or the 2013 ACR/EULAR classification criteria for adult SSc (additive weighted score, threshold ≥9 points) [19]. Since no firm classification criteria for JSSc overlap syndromes (OS) have been established and due to their clinical variability, patients with OS were excluded.

According with the recent classification proposal [6], we considered four subtypes of JSSc: limited cutaneous subtype (lcJSSc), diffuse cutaneous subtype (dcJSSc), systemic sclerosis sine-scleroderma subtype (ssJSSc), and the newly proposed fibrotic subtype (fJSSc), characterized by diffuse skin involvement, lack of both Raynaud’s phenomenon and SSc-specific autoantibodies [6, 13, 20, 21].

### Baseline variables and data collection

Demographics, clinical features, laboratory markers, scleroderma-specific antibodies, concomitant treatments and remission rates were captured in a harmonized protocol for collection of serial data previously reported in detail [6].

Data collected included: sex, age at diagnosis, Raynaud’s phenomenon (RP), digital ulcers (DU), cutaneous involvement assessed using the modified Rodnan Skin Score (mRSS), musculoskeletal involvement including presence of limited joint range of motion, tendon friction rubs or active arthritis or myositis, symptoms related to gastroesophageal reflux such as pyrosis and dysphagia, abnormal oesophageal transit, assessed by barium oesophagogram, oesophageal scintigraphy or manometry, respiratory involvement assessed by forced vital capacity (FVC) and diffusion lung capacity of carbon monoxide (DLCO), and chest high-resolution computed tomography (HRCT), cardiac involvement monitored by electrocardiogram (ECG), conventional or speckle tracking echocardiography (ECHO), systolic pulmonary arterial pressure (sPAP) quantified by standard echocardiography and, in selected cases, by right heart catheterization and the renal involvement including the persistent presence of proteinuria >0.5 g/day and/or functional impairment, with or without scleroderma renal crisis (SRC). The auto-antibody profiles included the assessment of antinuclear antibodies (ANA) and the main SSc-specific autoantibodies (anti-topoisomerase-I also knowns as anti-Scl-70, anti-centromere, anti-RNA-polymerase III).

According with the more recent recommendations for JSSc, patients were treated in a protocol-based manner with low-dose prednisone, methotrexate (MTX), mycophenolate mofetil (MMF) and/or rituximab (RTX) in the more severe cases [22]. Patients were followed up closely, at 3–4 months intervals during the first year. Depending on the response to treatment, visits were then spaced out to every 4–6 months.

The study was a secondary use study of previously collected clinical data, and no participant contact was required, meaning the requirement for written consent was waived. All data were anonymized and collected in accordance with Good Clinical Practice in a secure electronic database approved by the University Hospital of Padova Ethics Committee for Experimental Studies (Resolutions 399/387, 08.10.2012).

### Outcome measures

The primary outcome was the progression-free survival (PFS) defined as the percentage change in the Juvenile Systemic Sclerosis Severity score (J4S) between baseline and follow-up visits, assessed at least every six months or at the time of clinically significant worsening [23]. Changes relative to baseline were calculated using the formula: *[(J4S_timeN_ − J4S_time0_)/J4S_time0_] × 100*. A change exceeding ±25% from baseline was considered clinically significant, with increases indicating worsening and decreases indicating improvement.

### Statistical analysis

Baseline and demographic characteristics were summarized using descriptive statistics. Continuous variables were presented as median (interquartile range, IQR) and compared between JSSc subtypes using the Mann–Whitney *U* test or Kruskal–Wallis test, as appropriate. Categorical variables are presented as *n* (%) and compared using the chi-squared test or Fisher’s exact test.

To describe disease progression over time, Kaplan–Meier survival analysis was performed to estimate progression-free survival (PFS), defined as the time to a clinically significant change in J4S, and to compare progression among the different disease subtypes.

To explore the number of classes and trajectory shapes, we initially applied latent class trajectory modelling (LCTM) to identify subgroups of patients with similar underlying J4S trajectories across visits [24]. LCTM was used as an exploratory approach to identify potential trajectory patterns, while GMM provided confirmatory refinement and formal estimation of latent classes. An initial single-class linear mixed-effects model was first fitted and subsequently used to provide starting values for the mixture model [25]. Longitudinal trajectories of the J4S score were analysed using GMM to identify unobserved subgroups of patients exhibiting similar patterns of change over time. GMM was selected based on our clinical hypothesis that distinct disease course clusters may exist [24]. We applied GMM to determine the optimal number of latent trajectory classes of disease severity as measured by J4S. GMM extends conventional mixed-effects growth models by allowing for latent classes, each characterized by a distinct mean trajectory, while accounting for within-subject correlation through random effects [26].

Time was modelled as a continuous variable expressed in months from diagnosis (0, 6, 12, 18, 24, 30, 36, 42 and 48 months). Model estimation was performed using maximum likelihood as implemented in the *lcmm* package in R [25].

The optimal number of latent classes was determined by comparing candidate one- to four-class models on log-likelihood, AIC, BIC, entropy, posterior probabilities of class assignment, class size, parsimony and clinical interpretability [27]. Model stability was assessed by re-estimating the two-class solution 50 times with different random seeds/starting values, examining consistency of fit indices and class-size patterns. Posterior probabilities were computed for each patient, who was assigned to the class with the highest probability for descriptive/comparative analyses [24]. Model-estimated class-specific trajectories and 95% CIs were compared with observed mean J4S values to aid interpretation, and subsequent analyses assessed baseline characteristics and outcomes across classes.

An initial set of 18 candidate baseline clinical variables was considered (Supplementary Table S1). Seven were selected *a priori* based on clinical relevance to disease severity and prognosis, data completeness, sufficient variability and non-redundancy, in order to preserve parsimony given the small sample size. Baseline predictors of class membership were examined using a three-step GMM approach to account for classification uncertainty.

For clinical validation, Kaplan–Meier survival analyses were performed to compare time-to-event outcomes among the latent trajectory classes, with differences assessed using the log-rank test. The Kaplan–Meier curves for predefined disease subtypes were compared with those derived from the GMM-identified latent classes using concordance analysis, including Cohen’s kappa and overall agreement.

The GMM was estimated using maximum likelihood, accommodating missing data under the missing at random (MAR) assumption and retaining all available observations without imputing J4S scores.

Statistical significance was defined as *P* < 0.05. All analyses were performed using SPSS version 20.0 (SPSS Inc., Chicago, IL, USA) and R version 4.3.2 (packages: lcmm, tidyverse, dplyr, pROC) [28].

## Results

### Baseline characteristics

Of 45 JSSc patients seen over 20 years, 38 with follow-up longer than four years entered the study: median age at onset 10.8 years (IQR 7.8–12.9), 25/38 (66%) female, median follow-up 7.0 years (IQR 4.9–11.4).

At diagnosis, median mRSS was 10.5 (IQR 3.5–18.0); vascular involvement affected 32/38 (84.2%), gastrointestinal involvement 32/38 (84.2%), pulmonary involvement 16/38 (42.1%), cardiac involvement 7/38 (18.4%) and renal involvement 2/38 (5.3%, including one SRC) (Table 1).

**Table 1:** Study population.

	**Overall** ^a^ (*n* = 38)	**Diffuse** (*n* = 11)	**Limited** (*n* = 17)	**Sine** (*n* = 5)	**Fibrotic** (*n* = 5)
**Female sex, *n* (%)**	25 (66)	8 (77)	11 (65)	2 (40)	4 (80)
**Age at onset, years, median (IQR)**	10.8 (7.8–12.9)	12.4 (7.7–12.6)	9.8 (7.3–12.4)	14.1 (8.1–16.1)	8.7 (7.3–13.1)
**mRSS at diagnosis, median (IQR)**	10.5 (3.5–18.0)	18.0 (15.0–26.0)	5.0 (3.0–11.5)	0.0 (0.0–0.0)	25.0 (20.5–25.5)
**Vascular involvement, *n* (%)**	32 (84.2)	11 (100)	16 (94.1)	5 (100)	0 (0)
**Pulmonary involvement, *n* (%)**	16 (42.1)	8 (72.7)	4 (23.5)	2 (40.0)	2(40.0)
**Cardiac involvement, *n* (%)**	7 (18.4)	3 (27.3)	0 (0.0)	4 (80.0)	4 (80.0)
**Gastrointestinal involvement, *n* (%)**	32 (84.2)	11 (100)	13 (76.5)	4 (80.0)	4 (80.0)
**Renal involvement, *n* (%)**	2 (5.3)	2 (18.2)	0 (0.0)	0 (0.0)	0 (0.0)
**No. internal organs involvement, median (IQR)**					
** Diagnosis**	1.0 (1.0–2.0)	2.0 (2.0–2.0)	1.0 (1.0–1.0)	2.0 (1.5–2.5)	1.0 (0.5–2.0)
** 48 mo. F/U**	1.0 (0.0–2.0)	2.0 (1.0–3.0)	1.0 (0.0–1.0)	3.0 (1.0–3.0)	0.0 (0.0–0.5)
**J4S score, median (IQR)**					
** Diagnosis**	9.0 (5.0–13.3)	13.0 (11.0–18.0)	7.0 (4.5–13.5)	9.0 (6.5–12.0)	5.0 (4.5–8.5)
** 48 mo. F/U**	6.0 (3.0–15.0)	16.0 (12.0–21.0)	6.0 (2.0–9.0)	16.0 (5.5–22.8)	2.0 (1.0–3.0)
**Remission at 48, months, *n* (%)**					
** Clinical remission off medication**	12 (31.6)	0 (0.0)	9 (52.9)	0 (0.0)	3 (60.0)
** Clinical remission on medication**	15 (38.5)	3 (27.3)	8 (47.1)	2 (40.0)	2 (40.0)
** Active-progressive disease**	11 (28.9)	8 (72.7)	0 (0.0)	3 (60.0)	0 (0.0)
**Follow up in years, median (IQR)**	7.0 (4.9–11.4)	6.0 (3.4–11.2)	8.0 (5.4–12.7)	5.9 (3.3–9.6)	11.0 (4.0–11.9)

Baseline demographic, clinical, disease severity, and remission characteristics of the 38 children with JSSc included in the study, overall and by clinical subtype (diffuse, limited, sine scleroderma, fibrotic).

a Values are number or mean (% or ± SD), unless otherwise specified.

J4S: Juvenile Systemic Sclerosis Severity Score; mRSS: modified Rodnan Skin Score.

Median internal organs involved was 1.0 (IQR 1.0–2.0) at diagnosis and 1.0 (IQR 0.0–2.0) at 48 months, highest in dcJSSc (2.0) and ssJSSc (3.0); median J4S score was 9.0 (IQR 5.0–13.3) at diagnosis and 6.0 (IQR 3.0–15.0) at 48 months, again highest in dcJSSc and ssJSSc (both 16.0).

At 48 months, 12/38 (31.6%) had achieved remission off medication, 15/38 (39.5%) remission on medication and 11/38 (28.9%) still had active disease, predominantly dcJSSc and ssJSSc (Table 1).

### Different disease progression over time across JSSc subtypes

Disease progression over the four years of follow-up differed across the four JSSc subtypes. At diagnosis, most patients with fJSSc, lcJSSc or ssJSSc had J4S scores between 6 and 9, whereas the dcJSSc subtype averaged ∼14, reflecting its greater severity from onset. Over time, lcJSSc and fJSSc followed an almost parallel, gradually decreasing course, while ssJSSc increased progressively in severity, eventually reaching values similar to dcJSSc (Supplementary Fig. S1).

We then analysed progression-free survival (PFS) using Kaplan–Meier curves. The course was consistently benign in fJSSc, and lcJSSc as they declined only slightly, with about 80% of patients progression-free at 48 months. The dcJSSc and ssJSSc subtypes behaved differently: dcJSSc worsened early, mainly within the first 6–12 months, then stabilized, whereas ssJSSc changed little in the first six months but worsened progressively between 18 and 42 months. These differences were statistically significant: PFS was lower in ssJSSc than in lcJSSc (*P* = 0.010) and fJSSc (*P* = 0.013), with a trend towards significance for the fJSSc *vs* dcJSSc comparison (*P* = 0.087) (Fig. 1).

**Figure 1:**
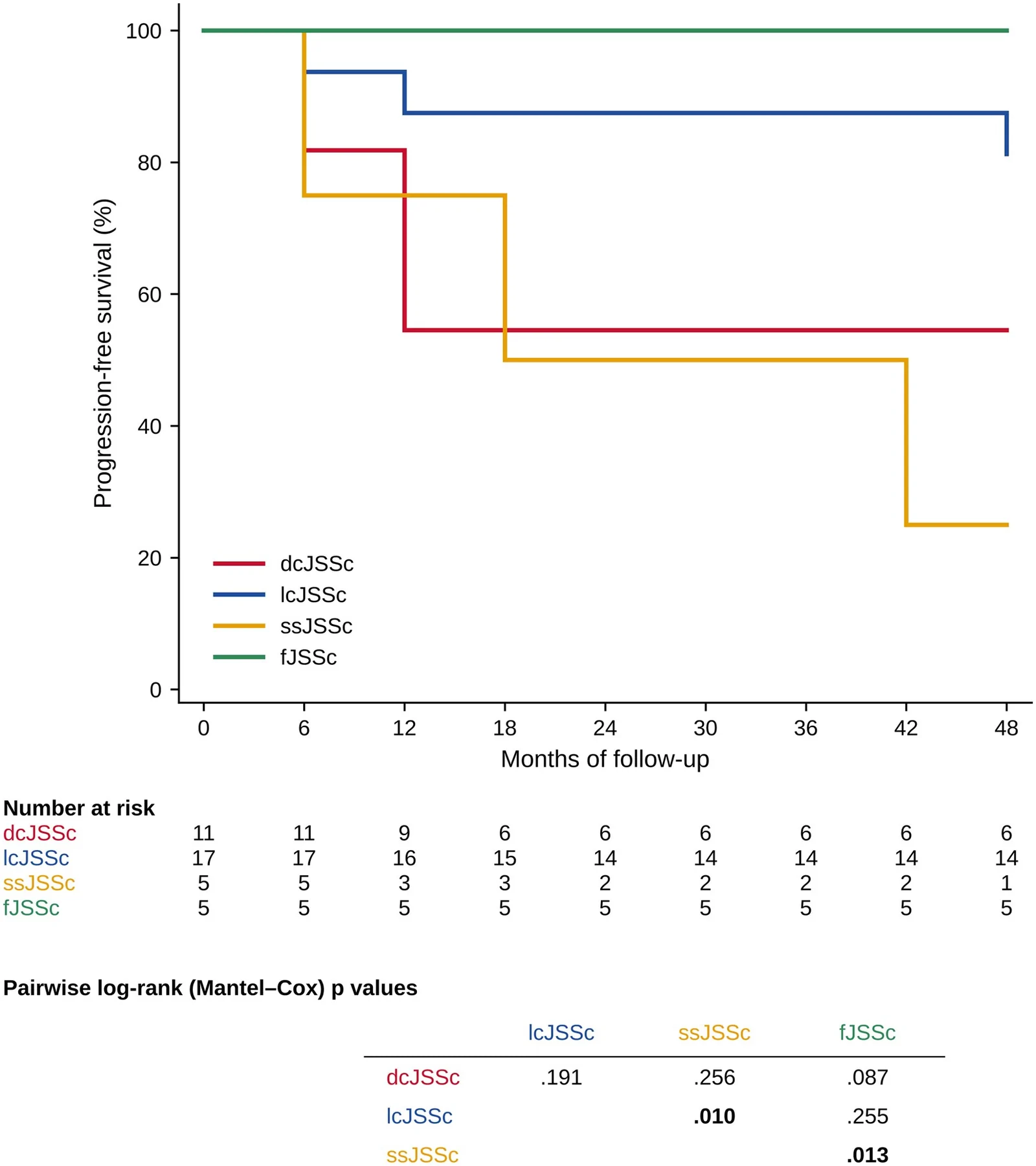
Progression-free survival over 48 months by the four JSSc clinical subtypes (*n* = 38). Kaplan–Meier estimates of progression-free survival in patients with diffuse cutaneous (dcJSSc) (red), limited cutaneous (lcJSSc) (blue), sine scleroderma (ssJSSc) (yellow) and fibrotic (fJSSc) (green) JSSc. Tick marks indicate censoring. Numbers at risk are shown below the *x*-axis. Comparison between groups has been done using the log-rank (Mantel–Cox) test; significant differences were observed between lcJSSc and ssJSSc (*P* = 0.010) and between ssJSSc and fJSSc (*P* = 0.013), whereas other comparisons were not significant

### Distinct longitudinal trajectories reveal latent severity subgroups in JSSc

Two latent classes of disease severity with distinct longitudinal trajectories based on additional variables other than or alternative to type of skin involvement or the characteristics of autoantibodies were identified.

Class 1 (74% of patients) comprised children with low disease severity at onset, good treatment response and sustained low severity over time; it included all children with lcJSSc and fJSSc, four with dcJSSc and two with ssJSSc. Class 2 (26%) followed an active, progressive course with a severe long-term outcome and included seven children with dcJSSc and three with ssJSSc (Table 2, Fig. 2). Observed and predicted trajectories agreed closely in Class 1; in Class 2 the predicted trajectory ran slightly worse than the observed one, most notably at 42 and 48 months (Fig. 2).

**Figure 2:**
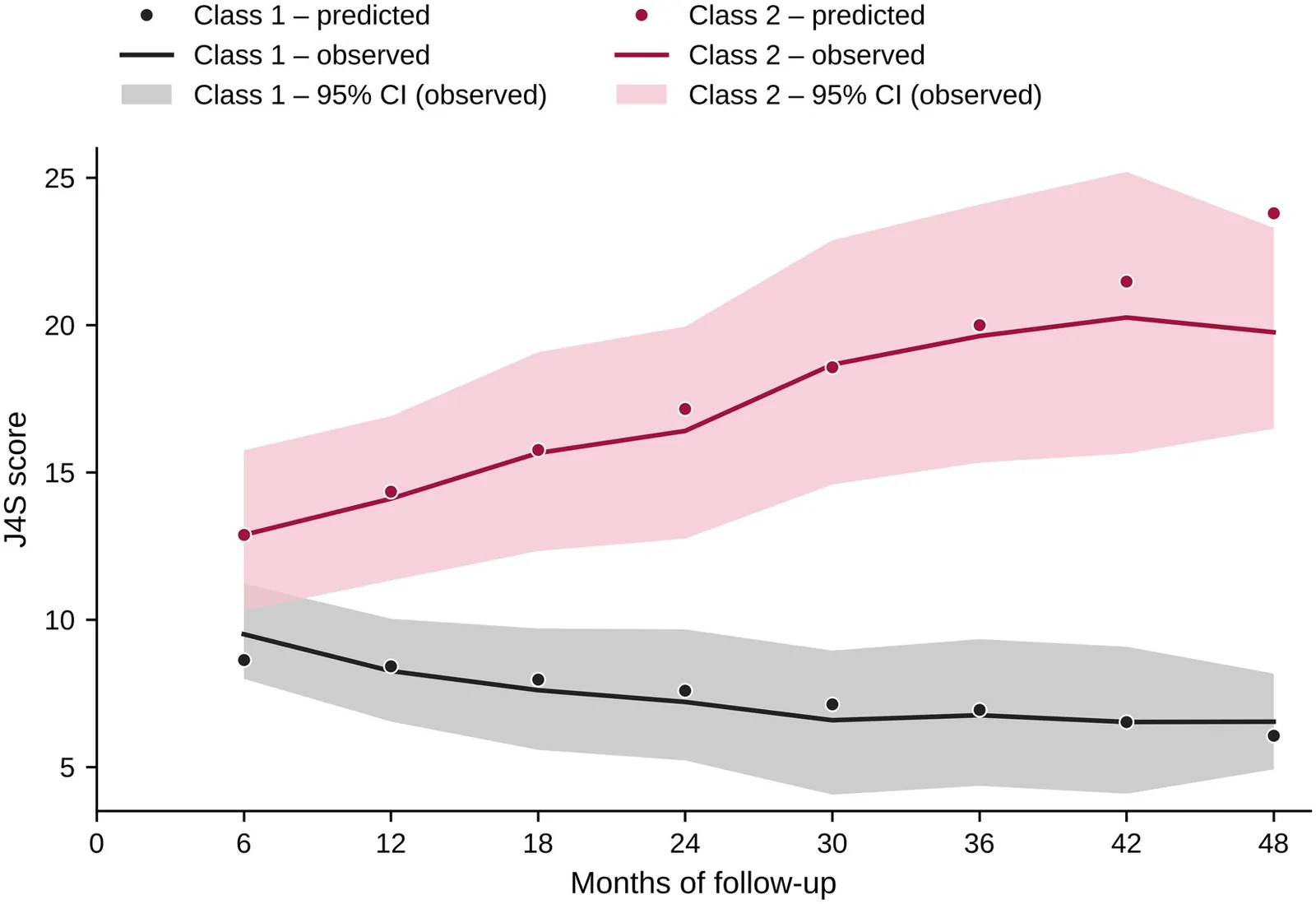
Observed *vs* predicted J4S score trajectories over 48 months by GMM-derived latent class (Class 1, *n* = 28; Class 2, *n* = 10). Observed (solid lines) and predicted (dots) J4S scores over time according to latent class membership. Shaded areas represent 95% CIs around the observed trajectories. Two distinct patterns were identified: class 1, characterized by lower baseline J4S scores and a decreasing or stable trajectory over time (grey), and class 2, characterized by higher baseline J4S scores with progressive worsening during follow-up (red). Time is shown in months. pred: GMM-predicted class trajectory; obs: observed class mean

**Table 2:** Variables associated with latent class membership.

	Class 1 (*n* = 28)	Class 2 (*n* = 10)	*P*-value
**Subtype, *n* (%)**			
** dcJSSc**	4 (36.4)	7 (63.6)	<0.001
** lcJSSc**	17 (100.0)	0 (0)	
** ssJSSc**	2 (40.0)	3 (60.0)	
** fJSSc**	5 (100.0)	0 (0)	
**J4S at diagnosis, mean (SD)**	9.0 (±4.3)	12.50 (±4.6)	0.053
**Number of internal organs involved, mean (SD)**	1.2 (0.6)	2.5 (0.9)	<0.001
**Pulmonary involvement, *n* (%)**	9 (32.1)	7 (70.0)	0.062
**Cardiac involvement, *n* (%)**	1 (3.6)	6 (60.0)	<0.001
**Disease duration at treatment initiation months, mean (SD)**	9.3 (16.2)	21.3 (19.0)	0.081
**Follow up years, mean (SD)**	8.8 (4.3)	6.4 (4.4)	0.091

Values are number (%), mean (±SD).

Comparison of the seven baseline variables retained in the Growth Mixture Model (GMM) between the two GMM-derived latent severity classes (Class 1, *n* = 28; Class 2, *n* = 10).

dcJSSc: diffuse cutaneous juvenile systemic sclerosis; lcJSSc: limited cutaneous juvenile systemic sclerosis; ssJSSc: sine-scleroderma juvenile systemic sclerosis; fJSSc: fibrotic juvenile systemic sclerosis; J4S: Juvenile Systemic Sclerosis Severity Score.

The optimal number of latent classes was determined by comparing GMM solutions with one to four classes (Supplementary Table S2): although BIC was lowest for the three-class solution, classification quality was highest for the two-class solution (entropy 0.916; mean posterior probability 0.982; no patient below 0.70, one below 0.80). Considering fit, classification quality, class size, parsimony and clinical interpretability, the two-class solution was retained. Stability was confirmed by 50 re-estimations (100% convergence; mean BIC 1710.53, SD 0.28; mean entropy 0.876, SD 0.049), with the two-class structure recovered in the majority of runs (Supplementary Table S3).

Seven of 18 candidate baseline variables screened (Supplementary Table S1) were selected *a priori*: disease subtype, J4S score at diagnosis, number of internal organs involved, pulmonary and cardiac involvement, disease duration at treatment initiation and follow-up time. These were not used to define the latent classes (derived solely from longitudinal J4S trajectories) but were examined for association with class membership (Table 2).

### Predictors of latent class membership

Using a three-step GMM approach to determine baseline predictors of latent class membership, three variables were identified: baseline J4S score (cut-off 7.5), disease duration at treatment initiation (cut-off 9 months) and number of internal organs involved (cut-off 1.5) (Table 3). These variables were significantly associated with class assignment across the GMM-derived trajectories and yielded moderate-to-high values for the area under the receiver operating characteristic (ROC) curve (AUC) in predicting cluster membership: baseline J4S score 0.71, disease duration at treatment initiation 0.68 and number of internal organs involved 0.90. Patients with values above these cut-offs were more likely to belong to Class 2, which was characterized by a worse clinical trajectory.

**Table 3:** Predictors of class membership.

Variable	Cut-off	Sensitivity	Specificity	AUC	95% CI
**J4S score at diagnosis**	7.5	0.90	0.50	0.71	(0.54–0.88)
**Disease duration at treatment initiation (months)**	9.0	0.67	0.74	0.68	(0.47–0.89)
**Number of internal organs involved**	1.5	1.00	0.71	0.90	(0.83–0.97)

Optimal cut-offs, sensitivity, specificity, and area under the ROC curve (AUC) for the continuous baseline variables significantly associated with latent class membership, identified using a three-step GMM approach.GMM: growth mixture modelling; J4S: Juvenile Systemic Sclerosis Severity Score; ROC: receiver operating characteristic.

### GMM-derived latent classes show distinct disease progression concordant with JSSc clinical subtypes

Kaplan–Meier analysis revealed distinct PFS patterns across the GMM-derived classes. Patients in latent Class 1 followed a largely benign course, with over 80% remaining progression-free at 48 months, reflecting the trends seen in lcJSSc and fJSSc. In contrast, patients in latent Class 2 experienced a more severe trajectory, with rapid deterioration between 6 and 18 months and further decline between 42 and 48 months, consistent with the course of dcJSSc and ssJSSc (Fig. 3). Comparing the Kaplan–Meier curves of the predefined clinical subtypes with those of the GMM-identified latent classes showed good concordance: 84.2% of patients were assigned to the same class in both classifications (κ = 0.66).

**Figure 3:**
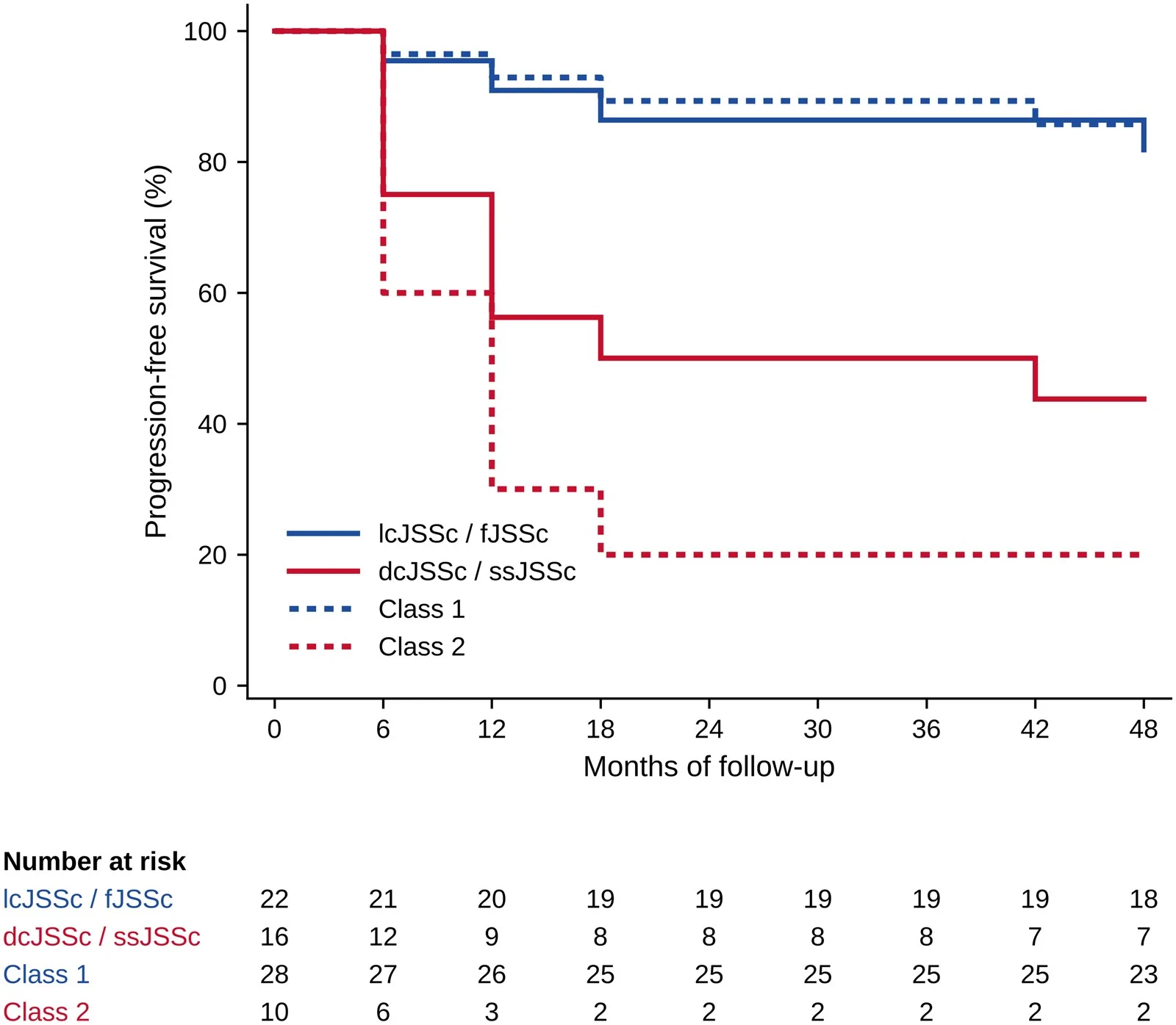
Concordance between progression-free survival by clinical subtype grouping and by GMM-derived latent class over 48 months. Kaplan–Meier estimates of progression-free survival stratified by combined clinical subtypes (lcJSSc/fiJSSc and dcJSSc/ssJSSc) (solid lines) and latent classes (Class 1 and Class 2) (dashed lines). Tick marks indicate censoring. Numbers at risk (with number censored) are shown below the *x*-axis. Patients in lcJSSc/fiJSSc and class 1 groups show more favourable progression-free survival compared with dcJSSc/ssJSSc and class 2 groups

## Discussion

This is the first study to identify two distinct latent subgroups of disease severity in children with JSSc using unsupervised machine learning (ML), and to demonstrate that high baseline J4S, treatment initiation beyond nine months of disease duration and greater internal organ involvement are the three key predictors of poor outcome.

During the last few years, artificial intelligence has been more and more adopted as an innovative tool for analysing high-dimensional, complex datasets, even within relatively small SSc cohorts [29].

A recent group-based trajectory modelling (GBTM) has shown that using only the baseline Scleroderma Clinical Trials Consortium Damage Index (SCTC-DI), cutaneous subset and sex were able to predict damage accrual in adult SSc [30]. Another study, again in adult patients, from the European Scleroderma Trials and Research (EUSTAR) database, using a mixed effects logistic regression models for organ involvement and Cox proportional hazards regression models, has shown that the autoantibody status is a better predictor of overall survival and disease progression over four years than is the cutaneous subtype [14]. For this reason, we explored how ML models can help assess disease course and build predictive models to inform personalized care also in JSSc.

First, we evaluated disease progression over time in different disease subtypes. Our analysis clearly showed that dcJSSc and ssJSSc subtypes are characterized by a significantly worse PFS than the other two forms, lcJSSc and fJSSc.

As for ssJSSc, we have already demonstrated that the greater involvement of internal organs, the absence of skin involvement and the resulting diagnostic delay play a role in the worse long-term prognosis [7]. Regarding dcJSSc, it appears to be similar, in many respects, to the adult forms, in which lung fibrosis, DU, increased C-reactive protein and muscle weakness are significantly related with increased mortality and morbidity [31]. The PFS of both limited and fibrotic JSSc subtypes was similar to what was observed in adults; however, in adult lcSSc, the higher frequency of DU and PAH, which are very rare in children, represent significant negative prognostic factors [31].

Overall, our findings provide clinical evidence for at least two distinct disease trajectories, with lcJSSc and fJSSc at the milder end of the spectrum and dcJSSc and ssJSSc at the more severe end.

We expanded our analysis by adopting an unsupervised ML model, capable of predicting a possible disease course based on multiple predictors other than or alternative to cutaneous subtypes or autoantibodies profile.

Two distinct latent trajectories were identified: Class 1 was characterized by low disease severity at onset, favourable response to treatment and good long-term outcomes, whereas Class 2 was defined by an active, progressive disease course associated with poorer outcomes. These two non-overlapping trajectories may predict class membership based on only three variables: baseline J4S score, disease duration at treatment initiation and number of internal organs involved at diagnosis.

Similar results were reported by the EUSTAR group using a semi-supervised Deep Learning model, which identified five clinical trajectories based on organ involvement and disease severity [32]. A mild-moderate group resembled our Class 1, consistent with the limited cutaneous forms comprising 60% of Class 1, while a severe group resembling Class 2 was subdivided into three clusters: a pale blue cluster, with predominant skin but moderate organ involvement, resembling fJSSc; a red cluster, with less severe skin but significant multiorgan progression, resembling the diffuse cutaneous form; and a novel dark blue cluster, with limited skin but frequent severe lung and heart involvement, resembling ssJSSc. A similar adult lcSSc subgroup with anti-topoisomerase antibodies and severe lung involvement was recently reported by Zanatta *et al.* [33]

Therefore, we identified two distinct latent classes of disease severity in children with JSSc that, in many respects, resemble those observed in adults with SSc, along with three key baseline predictors of class membership that enable early risk stratification and support tailored therapeutic strategies and optimized monitoring. Importantly, this is the first demonstration of latent trajectory modelling in a paediatric JSSc population, extending advanced analytical methodologies previously applied only in adult cohorts and providing a novel framework for risk stratification in this rare disease.

For clinical validation, Kaplan–Meier survival analyses were performed to compare time-to-event outcomes among the latent trajectory classes. Interestingly, the two distinct GMM-derived classes closely mirrored the groups previously identified by the PFS analysis, with dcJSSc and ssJSSc on one end and lcJSSc and fJSSc on the other. Consequently, Kaplan–Meier curves for the predefined clinical groups were compared with those of the GMM-derived classes, demonstrating a similar separation between groups and good concordance, and suggesting that the latent trajectory classes reflect clinically meaningful patterns of disease severity. A greater discrepancy was observed between the dcJSSc/ssJSSc group and Class 2, which otherwise exhibited a more severe course, as six patients in the dcJSSc/ssJSSc group (four with dcJSSc and two with ssJSSc) instead showed a more benign course and were classified in Class 1.

Thus, the newly identified latent classes (Class 1 and Class 2) demonstrate clear prognostic significance by capturing distinct disease severity trajectories and aligning with previously suspected clinical classifications, thereby reinforcing the validity of our original subtype-based grouping.

Validity of the two latent classes was supported both internally and externally. Internally, entropy (0.916) and mean posterior probability of class assignment (0.982) indicated well-separated, stable classes. Externally, the classes showed strong concordance with pre-existing clinical JSSc subtypes (84.2% agreement, κ = 0.66) and differential association with progression-free survival on Kaplan–Meier analysis. This convergence between the data-driven, trajectory-based classification and established clinical subtyping supports the biological plausibility of the latent classes and suggests added prognostic value beyond conventional subtyping by identifying higher- and lower-risk patients within a given clinical subtype.

Our study has some limitations. First, this single-centre design, chosen to limit interobserver variation, yielded a relatively small sample size that may limit generalizability, reduce power to detect subtle between-subgroup differences and constrain the number of latent classes and precision of trajectories identified by GMM/LCTM. Second, excluding overlap syndromes, though necessary for cohort homogeneity, may limit applicability to the broader JSSc population. Third, as with adult SSc, no single diagnostic test exists for JSSc; patients were identified using validated classification criteria rather than a diagnostic gold standard, and the 20-year recruitment period may have introduced diagnostic and imaging heterogeneity over time. External validation in independent cohorts is therefore warranted.

In conclusion, we describe, for the first time, progression-free survival in a monocentric cohort of patients with JSSc with prolonged, standardized follow-up. Using an unsupervised machine learning model, we identified two longitudinal patterns of disease severity in JSSc. A high baseline J4S score, delayed treatment initiation and a greater number of internal organs involved at disease onset predicted a more severe disease course. Ultimately, integrating clinical and machine learning-based insights paves the way for patient-centred precision health strategies, with the potential to improve long-term outcomes through proactive, individualized disease management.

## Supplementary Material

keag584_Supplementary_Data

## Data Availability

Written requests for access to de-identified data published in this Article may be submitted to the corresponding author and will be adjudicated by the study data access committee.
