# Supplementary material for: Distinct trajectories of disease severity progression in juvenile systemic sclerosis
Source: Rheumatology (Oxford). 2026 Oct 20;65(11):keag584. doi: 10.1093/rheumatology/keag584 (PMC13738805; doi:10.1093/rheumatology/keag584)
Supplement: Supplementary file 1 [file keag584_supplementary_data.zip › rhe-26-1174-File008.docx]

Table of Contents

[Table of Contents	1](#_Toc236308959)

[Supplementary Figures	2](#_Toc236308960)

[**Supplementary Figure S1: Disease progression over time by juvenile Systemic Sclerosis subtype**	2](#_Toc236308961)

[Supplementary Tables	3](#_Toc236308962)

[**Supplementary Table S1: Distribution of variables by latent class**.	3](#_Toc236308963)

[**Supplementary Table S2**: **Model fit statistics for candidate latent class solutions (k = 1–4)**	5](#_Toc236308964)

[**Supplementary Table S3: Stability of the two-class GMM solution across repeated model estimations**	6](#_Toc236308965)

# **Supplementary Figures**

## **Supplementary Figure S1: Disease progression over time by juvenile Systemic Sclerosis subtype.** Mean J4S scores over 48 months of follow-up in patients with diffuse cutaneous (dcJSSc) (red), limited cutaneous (lcJSSc) (blue), sine scleroderma (ssJSSc) (yellow), and fibrotic (fJSSc) (green) JSSc. Lines represent mean values at each timepoint.

# **Supplementary Tables**

## **Supplementary Table S1: Distribution of variables by latent class.** Distribution of the 18 baseline variables screened for the GMM, by latent class (Class 1, n=28; Class 2, n=10). Values are presented as appropriate for variable type.

|  | | **Class 1**  No. 28 | **Class 2**  No. 10 | **p-value** |
| --- | --- | --- | --- | --- |
| **Sex** (female) | | 19 (67.9%) | 6 (60.0%) | 0.71 |
| **Age at onset** | median | 10.4 (7.8-12.5) | 11.5 (8.1-13.1) | 0.56 |
|  | mean | 10.1 (±3.3) | 10.8 (±3.7) |  |
| **Subtype** | dcJSSc | 4 (36.4%) | 7 (63.6%) | **<0.001** |
|  | lcJSSc | 17 (100.0%) | 0 (0%) |  |
|  | ssJSSc | 2 (40.0%) | 3 (60.0%) |  |
|  | fJSSc | 5 (100.0%) | 0 (0%) |  |
| **J4S at diagnosis** | median | 7.5 (5.0-13.0) | 12.0 (9.0-14.5) | 0.053 |
|  | mean | 9.0 (±4.3) | 12.5 (±4.7) |  |
| **mRSS at diagnosis** | median | 10.5 (4.0-19.3) | 12.5 (2.5-17.5) | 0.80 |
|  | mean | 12.2 (±10.7) | 10.7 (±8.1) |  |
| **SSc-specific autoantibodies** | | 12 (42.9%) | 7 (70.0%) | 0.27 |
| **NVC pattern active-late** | | 20 (71.4%) | 7 (70.0%) | 1.0 |
| **Number of internal organs involved** | median | 1.0 (1.0-2.0) | 2.0 (2.0-2.8) | **<0.001** |
|  | mean | 1.2 (±0.6) | 2.5 (±0.9) |  |
| **Vascular involvement** | | 22 (78.6%) | 10 (100.0%) | 0.17 |
| **Pulmonary involvement** | | 9 (32.1%) | 7 (70.0%) | 0.062 |
| **Cardiac involvement** | | 1 (3.6%) | 6 (60.0%) | **<0.001** |
| **Gastrointestinal involvement** | | 22 (78.6%) | 10 (100.0%) | 0.17 |
| **Renal involvement** | | 0 (0.0%) | 2 (20.0%) | 0.064 |
| **Therapy** | | MTX: 18 (66.7%) \|  MMF: 9 (33.3%) | MTX: 3 (33.3%)\|  MMF: 6 (66.7%) | 0.12 |
| **Disease duration at treatment initiation in months** | median | 0.0 [0.0-9.0] | 24.00 [0.0-36.0] | 0.081 |
|  | mean | 9.3 (±16.2) | 21.3 (±19.0) |  |
| **Treatment duration** (months) | median | 48.0 (36.0-75.0) | 42.0 (30.0-60.0) | 0.19 |
|  | mean | 56.7 (±24.3) | 44.0 (±24.7) |  |
| **Follow up in years** | median | 8.2 (5.5-11.7) | 5.70 (3.7-7.7) | 0.091 |
|  | mean | 8.8 (±4.3) | 6.4 (±4.4) |  |
| **Active-progressive disease** | | 2 (7.1%) | 10 (100.0%) | **<0.001** |

Values are number (%), median (95% CI), mean (±SD)

Abbreviation: J4S: J4S: Juvenile Systemic Sclerosis Severity score; mRSS: modified Rodnan Skin Score; NVC: nailfold videocapillaroscopy; MTX: methotrexate; MMF: mycophenolate mofetil.

## **Supplementary Table S2**: **Model fit statistics for candidate latent class solutions (k = 1–4)**. Growth Mixture Models with one to four latent classes were fitted to the longitudinal J4S trajectories and compared on model fit, classification quality, and class size.

| **k** | **Log-likelihood** | **Parameters, n** | **AIC** | **BIC** | **Entropy** | **Posterior probability, mean (median; min)** | **n <0.70 / n <0.80** | **Class sizes, n (%)** |
| --- | --- | --- | --- | --- | --- | --- | --- | --- |
| 1 | −848.58 | 6 | 1709.15 | 1718.98 | n/a | n/a | n/a | C1=38 (100%) |
| **2*** | **−837.19** | **10** | **1694.38** | **1710.76** | **0.916** | **0.982 (0.999; 0.743)** | **0 / 1** | **C1=10 (26.3%); C2=28 (73.7%)** |
| 3 | −825.62 | 14 | 1679.23 | 1702.16 | 0.843 | 0.944 (0.961; 0.569) | 1 / 2 | C1=8 (21.1%); C2=14 (36.8%); C3=16 (42.1%) |
| 4 | −821.94 | 18 | 1679.88 | 1709.36 | 0.879 | 0.947 (0.979; 0.573) | 1 / 3 | C1=8 (21.1%); C2=9 (23.7%); C3=14 (36.8%); C4=7 (18.4%) |

Abbreviations: AIC, Akaike Information Criterion; BIC, Bayesian Information Criterion; J4S, Juvenile Systemic Sclerosis Severity score.

## **Supplementary Table S3: Stability of the two-class GMM solution across repeated model estimations.** The two-class model was re-estimated 50 times using different random seeds and starting values to assess the stability of model fit and class assignment.

| **Runs, n** | **Converged, n (%)** | **Mean BIC (SD)** | **BIC range** | **Mean log-likelihood (SD)** | **Mean entropy (SD)** | **Class-size patterns observed** |
| --- | --- | --- | --- | --- | --- | --- |
| 50 | 50 (100%) | 1710.53 (0.28) | 1710.19–1710.76 | −837.08 (0.14) | 0.876 (0.049) | 28/10 (majority of runs); 10/28 (same solution, reversed labels); 24/14 (minority of runs) |

Abbreviations: BIC, Bayesian Information Criterion; SD, standard deviation.
